# Colorectal cancer as a complex adaptive system: integrating the hallmarks of cancer with complexity theory

**DOI:** 10.3389/fonc.2025.1736140

**Published:** 2026-02-03

**Authors:** Luis R. Basbus

**Affiliations:** Department of Oncology, Hospital Italiano de Buenos Aires, Buenos Aires, Argentina

**Keywords:** colorectal cancer, complex systems, emergence, hallmarks of cancer, self-organization, systems biology

## Abstract

The paradigm of the *Hallmarks of Cancer*, updated by Douglas Hanahan in 2022, represents one of the most influential syntheses for understanding the functional capabilities that sustain neoplastic transformation. However, its traditional interpretation, often reductionist and fragmentary, does not capture the non-linear, emergent, and adaptive dynamics of tumor behavior. This review proposes a reinterpretation of the hallmarks through the lens of complexity theory, conceptualizing colorectal cancer (CRC) as a self-organizing, open system operating far from equilibrium. Using an integrative conceptual approach, we map the ten classical hallmarks and the new dimensions proposed in 2022 (phenotypic plasticity, non-mutational epigenetic reprogramming, polymorphic microbiomes, and senescence) onto the fundamental properties of complex systems: nonlinearity, emergence, feedback, openness, and historical dependence. We argue that CRC should not be understood as a simple sum of molecular alterations but as a dynamic network of interactions among cells, tissues, and microenvironments where global organization emerges from local rules. This systems-based perspective provides a conceptual foundation for translational models and integrative methodologies in oncology.

## 1 Introduction

For decades, cancer has been understood within a reductionist epistemology, as a sequence of genetic alterations that activate oncogenes, inactivate tumor suppressors, and drive uncontrolled proliferation (1). This model enabled key discoveries in molecular oncology but fails to explain emergent phenomena such as tumor heterogeneity, phenotypic plasticity, therapy resistance, and clonal evolution (2).

Hanahan’s 2022 update of the *Hallmarks of Cancer* expanded the original framework to include new enabling dimensions: non-mutational epigenetic reprogramming, polymorphic microbiomes, cellular senescence, and phenotypic plasticity (3). This conceptualization reframes cancer as a dynamic, evolving system in which biological capabilities emerge from multilevel interactions among genetic, epigenetic, and environmental components. Hanahan himself emphasizes that the hallmarks are heuristic tools for distilling the vast complexity of cancer phenotypes into a set of underlying organizational principles (3).

From this view, the tumor is not merely a mass of mutated cells but a **complex adaptive system** composed of interacting subsystems: epithelial, stromal, immune, and microbial elements that coevolve with their environment (4). Complexity theory, developed from systems physics, network theory, and ecology, privileges interactions over isolated entities, emergence over linear causality, and historicity over repetition (5). Applied to colorectal cancer, this framework positions the tumor as a dynamic ecosystem sustained by feedback loops, cooperation, and competition among its cellular and molecular components (6).

## 2 Methods

An integrative conceptual review was conducted to synthesize Hanahan’s 2022 hallmarks framework with literature on complex systems theory, tumor ecology, and colorectal carcinogenesis. The approach was explicitly non-systematic but analytically structured.

Relevant sources were identified through purposive searches in PubMed and Google Scholar, complemented by backward and forward citation tracking from seminal papers in cancer systems biology, complexity science, and colorectal cancer. Priority was given to: (i) highly cited conceptual and theoretical articles on cancer as a complex adaptive system; (ii) reviews and perspectives on tumor ecosystems, multiscale modeling, and systems oncology; and (iii) key empirical and translational works in colorectal cancer that illustrate emergent, network-level behaviors rather than isolated molecular events.

For the conceptual mapping, each hallmark and enabling characteristic was analyzed using predefined questions (1): Which interactions (cell–cell, cell–microenvironment, cell–microbiome) are necessary for this hallmark to manifest? (2) Which core properties of complex systems (nonlinearity, emergence, feedback, openness, interdependence, historical dependence) are required to sustain it? (3) How does the hallmark contribute to system-level adaptation or state transitions? Based on these criteria, hallmarks were iteratively assigned to one or more complexity properties, with emphasis on parsimony and internal coherence rather than exhaustive categorization. The objective was not to re-interpret primary molecular data but to construct a coherent systemic ontology of cancer grounded in established theoretical and experimental literature.

## 3 Results

### 3.1 The colorectal tumor as a complex adaptive system

Colorectal cancer represents a multilevel system composed of genetically unstable epithelial cells, stromal elements, immune infiltrates, vascular networks, and microbiota, all interacting within a dynamic intestinal environment (7). Global behavior emerges from local interactions and feedback mechanisms that cannot be predicted from any single component. The tumor behaves as a self-organizing structure, maintaining metastable states far from equilibrium through continuous energy and information exchange (8).

### 3.2 Mapping the hallmarks to complexity properties

Each hallmark of cancer reflects a manifestation of systemic dynamics rather than isolated molecular events. Sustained proliferative signaling arises from self-reinforcing feedback circuits, such as WNT–MAPK–EGFR cascades, which function as dynamic attractors in non-linear systems (9). Evading growth suppressors involves collective reorganization of tissue architecture, as loss of SMAD4 or APC disrupts epithelial containment and triggers adaptive pattern formation (10).

Resistance to cell death exemplifies system-level stabilization through positive feedback loops involving p53, NF-κB, BCL2, and HIF-1α (11). Replicative immortality reflects the open, dissipative nature of tumors that maintain energy fluxes to resist entropy (12). Induced angiogenesis operates as a recursive feedback process driven by HIF-1α and VEGF signaling under hypoxia (13).

Invasion and metastasis emerge as collective behaviors resulting from coordinated cell–matrix–immune interactions (14). Metabolic reprogramming underscores the system’s openness, allowing adaptive exchanges of energy and metabolites with the host (15). Immune evasion, finally, represents a dynamic coevolutionary process between tumor and immune system (16).

The enabling characteristics, genomic instability and tumor-promoting inflammation, sustain adaptation by introducing variability and historical memory into the system (17). Together, these hallmarks define cancer not as a collection of traits but as an emergent organization shaped by nonlinearity and feedback.

The conceptual mapping between hallmarks and complexity-theory properties is summarized in **Table 1**. For each hallmark, the table indicates the dominant systemic properties involved (e.g., nonlinearity, emergence, feedback, openness, historical dependence) and provides a concise explanation of how these properties manifest in colorectal cancer as a complex adaptive system.

**Table 1:** Mapping of Hanahan’s hallmarks of cancer (2022 update) onto core properties of complex adaptive systems in colorectal cancer.

Hallmark/new dimension (Hanahan 2022)	Dominant complexity properties	Conceptual link in colorectal cancer as a complex adaptive system
Sustaining proliferative signaling	Nonlinearity; feedback; emergence	WNT–MAPK–EGFR self-reinforcing loops act as dynamic attractors where small perturbations generate disproportionate proliferative shifts.
Evading growth suppressors	Interdependence; emergence; historical dependence	APC/SMAD4 disruption alters epithelial architecture, producing emergent dysplastic patterns not explainable by single-gene effects.
Resisting cell death	Feedback; nonlinearity; historical dependence	Interacting p53–NF-κB–BCL2–HIF-1α networks generate multistable survival regimes modulated by local stress conditions.
Enabling replicative immortality	Openness; dissipative structure; historical dependence	Telomere maintenance and sustained energy flux allow persistence far from equilibrium, embedding evolutionary memory.
Inducing angiogenesis	Feedback; openness; interdependence	Hypoxia-driven HIF-1α/VEGF signaling couples metabolic demand to vascular remodeling in co-evolving spatial niches.
Activating invasion and metastasis	Emergence; interdependence; nonlinearity	Collective migration and matrix remodeling arise from coordinated epithelial–stromal–immune interactions.
Deregulating cellular energetics	Openness; feedback; emergence	Warburg-like metabolic shifts reflect adaptive redistribution of energy across tumor–stroma–host interfaces.
Avoiding immune destruction	Coevolution; feedback; historical dependence	Iterative selection between tumor clones and immune infiltrates generates hot/altered/cold immune states.
Genome instability and mutation (enabling characteristic)	Historical dependence; variability generation	Ongoing instability expands the system’s evolutionary state space, increasing adaptability under therapy.
Tumor-promoting inflammation (enabling characteristic)	Feedback; openness; emergence	Cytokine–stromal–epithelial loops reinforce pro-tumor attractor states and microenvironmental restructuring.
Phenotypic plasticity (new hallmark)	Nonlinearity; multistability; emergence	EMT–MET transitions represent shifts between attractor states enabling rapid adaptation to selective pressures.
Non-mutational epigenetic reprogramming (new hallmark)	Historical dependence; network reconfiguration	Epigenetic remodeling alters regulatory topology, storing environmental information without changing DNA sequence.
Polymorphic microbiomes (new hallmark)	Openness; interdependence; feedback	Microbiome–immune–metabolic coupling reshapes the fitness landscape for tumor evolution.
Senescence and SASP (new hallmark)	Historical dependence; feedback; emergence	Senescent cells act as slow nodes releasing SASP mediators that restructure niches and modulate state transitions.

### 3.3 New dimensions as expressions of complexity

The new dimensions proposed by Hanahan (3) deepen this systemic reading. Phenotypic plasticity corresponds to transitions between attractor states within the tumor’s dynamic landscape (3). Non-mutational epigenetic reprogramming alters network topology without changing DNA sequence, conferring adaptability and memory (18). The microbiome acts as an open coupled environment influencing inflammation, immunity, and metabolism (18, 19). Senescent cells function as “slow nodes,” releasing inflammatory mediators that reshape the microenvironment and modulate state transitions (20).

## 4 Discussion

Colorectal cancer, when seen through the lens of complexity, ceases to be a sum of mutations and becomes an adaptive network governed by universal principles: emergence, feedback, self-organization, and openness. This paradigm explains heterogeneity and therapeutic resistance not as failures of precision medicine but as inevitable consequences of living systems capable of adaptation. It encourages the integration of multiscale models, agent-based simulations, and network approaches that incorporate molecular, ecological, and temporal data.

Understanding CRC as a complex system reframes therapy as ecological modulation rather than eradication. Targeting intercellular communication, metabolic cooperation, and microenvironmental dependencies may prove more effective than inhibiting single pathways. This shift aligns oncology with systems biology and network science, unifying the molecular and ecological dimensions of cancer.

### 4.1 Clinical translation

Understanding colorectal cancer as a complex adaptive system has important therapeutic implications. This framework shifts the focus from targeting isolated molecular pathways toward modulating system-level interactions that sustain tumor organization. From a therapeutic standpoint, complexity theory supports strategies that disrupt key ecological relationships—such as metabolic cooperation, stromal–epithelial feedback, and immune–tumor coevolution—rather than exclusively inhibiting single driver alterations. It also encourages adaptive and evolution-informed treatment designs, in which therapy is guided by anticipated state transitions, resistance trajectories, and the stabilization of less aggressive attractor states. Furthermore, integrating microbiome composition, immune contexture and spatial tissue organization into clinical decision-making may enable more robust stratification than purely genomic profiling. Finally, acknowledging the nonlinear and emergent nature of tumor behavior underscores the value of multiscale, longitudinal data and computational modeling as complementary tools for predicting responses and tailoring interventions. Taken together, this conceptual framework offers a path toward more resilient, ecologically informed therapeutic strategies in colorectal cancer.

## 5 Conclusions

This systems-oriented interpretation of colorectal cancer is consistent with advances in network biology, multiscale modeling, and ecological approaches to cancer, which conceptualize tumors as dynamic, interacting networks rather than linear molecular cascades (21–25). Recent work in cancer systems medicine and patient-specific mechanistic modeling further supports the integration of computational, ecological, and evolutionary frameworks to capture tumor behavior across scales (26–30).

Colorectal cancer should be conceived as a complex adaptive system where hallmarks emerge from collective organization rather than from discrete molecular events. The new dimensions proposed by Hanahan do not merely expand the list of hallmarks; they redefine cancer as an ecological, evolutionary, and self-organizing process. Embracing complexity offers not only a deeper theoretical understanding but also a pathway toward predictive and integrative oncology.

## Data Availability

The original contributions presented in the study are included in the article/supplementary material. Further inquiries can be directed to the corresponding author.

## References

[1] Fearon ER Vogelstein B . A genetic model for colorectal tumorigenesis. Cell. (1990) 61:759–67., PMID: 2188735 10.1016/0092-8674(90)90186-i

[2] Greaves M Maley CC . Clonal evolution in cancer. Nature. (2012) 481:306–13. 10.1038/nature10762 PMC3367003 22258609

[3] Hanahan D . Hallmarks of cancer: new dimensions. Cancer Discov. (2022) 12:31–46. doi: 10.1158/2159-8290.CD-21-1059, PMID: 35022204

[4] Quail DF Joyce JA . Microenvironmental regulation of tumor progression and metastasis. Nat Med. (2013) 19:1423–37. doi: 10.1038/nm.3394, PMID: 24202395 PMC3954707

[5] Bar-Yam Y . Dynamics of complex systems. Reading: Addison-Wesley (1997).

[6] Merlo LMF Pepper JW Reid BJ Maley CC . Cancer as an evolutionary and ecological process. Nat Rev Cancer. (2006) 6:924–35. doi: 10.1038/nrc2013, PMID: 17109012

[7] Baghban R Roshangar L Jahanban-Esfahlan R Seidi K Ebrahimi-Kalan A Jaymand M . Tumor microenvironment complexity and therapeutic implications at a glance. Cell Commun Signal. (2020) 18:59. doi: 10.1186/s12964-020-0530-4, PMID: 32264958 PMC7140346

[8] Prigogine I Stengers I . The new alliance: metamorphosis of science. Madrid: Alianza Editorial (1983).

[9] Segditsas S Tomlinson I . Colorectal cancer and genetic alterations in the Wnt pathway. Oncogene. (2006) 25:7531–7., PMID: 17143297 10.1038/sj.onc.1210059

[10] Becht E de Reyniès A Giraldo NA Pilati C Buttard B Lacroix L . Immune and stromal classification of colorectal cancer and its clinical relevance. Clin Cancer Res. (2016) 22:4057–66. doi: 10.1158/1078-0432.CCR-15-2879, PMID: 26994146

[11] Vousden KH Lane DP . p53 in health and disease. Nat Rev Mol Cell Biol. (2007) 8:275–83. doi: 10.1038/nrm2147, PMID: 17380161

[12] Carmeliet P Jain RK . Principles and mechanisms of vessel normalization for cancer and other angiogenic diseases. Nat Rev Drug Discov. (2011) 10:417–27. doi: 10.1038/nrd3455, PMID: 21629292

[13] Lambert AW Pattabiraman DR Weinberg RA . Emerging biological principles of metastasis. Cell. (2017) 168:670–91. 10.1016/j.cell.2016.11.037 PMC5308465 28187288

[14] Vander Heiden MG Cantley LC Thompson CB . Understanding the Warburg effect: the metabolic requirements of cell proliferation. Science. (2009) 324:1029–33., PMID: 19460998 10.1126/science.1160809 PMC2849637

[15] Galon J Bruni D . Approaches to treat immune hot, altered and cold tumours with combination immunotherapies. Nat Rev Drug Discov. (2019) 18:197–218. doi: 10.1038/s41573-018-0007-y, PMID: 30610226

[16] Burrell RA McGranahan N Bartek J Swanton C . The causes and consequences of genetic heterogeneity in cancer evolution. Nature. (2013) 501:338–45. 10.1038/nature12625 24048066

[17] Baylin SB Jones PA . Epigenetic determinants of cancer. Cold Spring Harb Perspect Biol. (2016) 8:a019505. doi: 10.1101/cshperspect.a019505, PMID: 27194046 PMC5008069

[18] Garrett WS . Cancer and the microbiota. Science. (2015) 348:80–6. 10.1126/science.aaa4972 PMC5535753 25838377

[19] Schwabe RF Jobin C . The microbiome and cancer. Nat Rev Cancer. (2013) 13:800–12. doi: 10.1038/nrc3610, PMID: 24132111 PMC3986062

[20] Newman MEJ . Networks: an introduction. Oxford: Oxford University Press (2010).

[21] Barabási AL Oltvai ZN . Network biology: understanding the cell’s functional organization. Nat Rev Genet. (2004) 5:101–13. 10.1038/nrg1272 14735121

[22] Vogelstein B Papadopoulos N Velculescu VE Zhou S Diaz LA Kinzler KW . Cancer genome landscapes. Science. (2013) 339:1546–58. 10.1126/science.1235122 PMC3749880 23539594

[23] Campisi J . Aging, cellular senescence, and cancer. Annu Rev Physiol. (2013) 75:685–705. doi: 10.1146/annurev-physiol-030212-183653, PMID: 23140366 PMC4166529

[24] Coppé JP Desprez PY Krtolica A Campisi J . The senescence-associated secretory phenotype: the dark side of tumor suppression. Annu Rev Pathol. (2010) 5:99–118. doi: 10.1146/annurev-pathol-121808-102144, PMID: 20078217 PMC4166495

[25] Swanton C . Embracing cancer complexity: hallmarks of systemic disease. Cell. (2024) 187:479–502. doi: 10.1016/j.cell.2024.01.003, PMID: 38552609 PMC12077170

[26] Karimi MR Hosseini M Biglar M Ramezani M Zali A Rezaei N . Prospects and challenges of cancer systems medicine. Brief Bioinform. (2022) 23:bbab343. doi: 10.1093/bib/bbab343, PMID: 34471925 PMC8769701

[27] Lorenzo G Ayensa-Jiménez J Gómez H Doblare M Cegonino J Martínez MA . Patient-specific mechanistic models of tumor growth and treatment. Annu Rev BioMed Eng. (2024) 26:1–27. doi: 10.1146/annurev-bioeng-082420-124839 38594947

[28] Aguadé-Gorgorió G Yan J Gore J . Modeling tumors as complex ecological systems. iScience. (2024) 27:108123. doi: 10.1016/j.isci.2024.108123 PMC11402243 39280631

[29] Wang J Wang E Chen L . Data-driven universal insights into tumorigenesis through hallmark-based gene regulatory networks. NPJ Syst Biol Appl. (2025) 11:12–25. doi: 10.1038/s41540-025-00463-1 41261114 PMC12630751

[30] Pavlova NN Thompson CB . The emerging hallmarks of cancer metabolism. Cell Metab. (2016) 23:27–47. doi: 10.1016/j.cmet.2015.12.006, PMID: 26771115 PMC4715268

